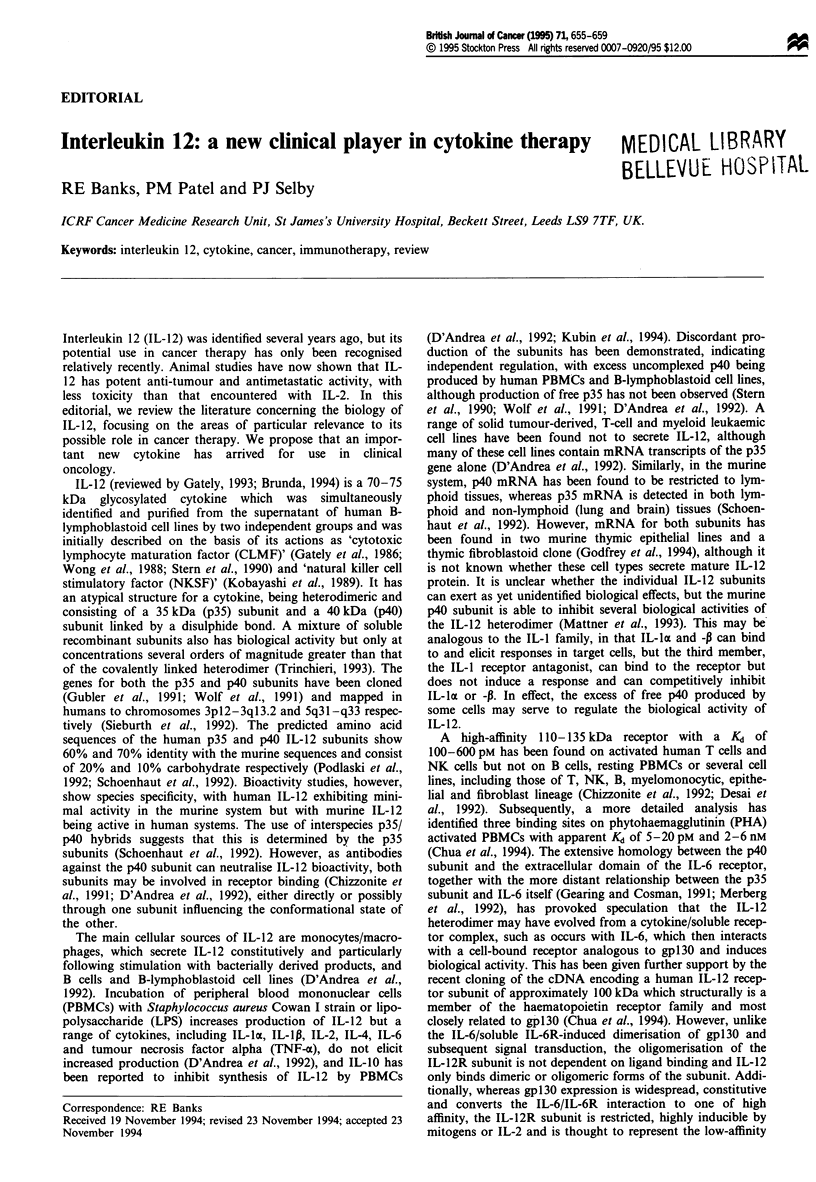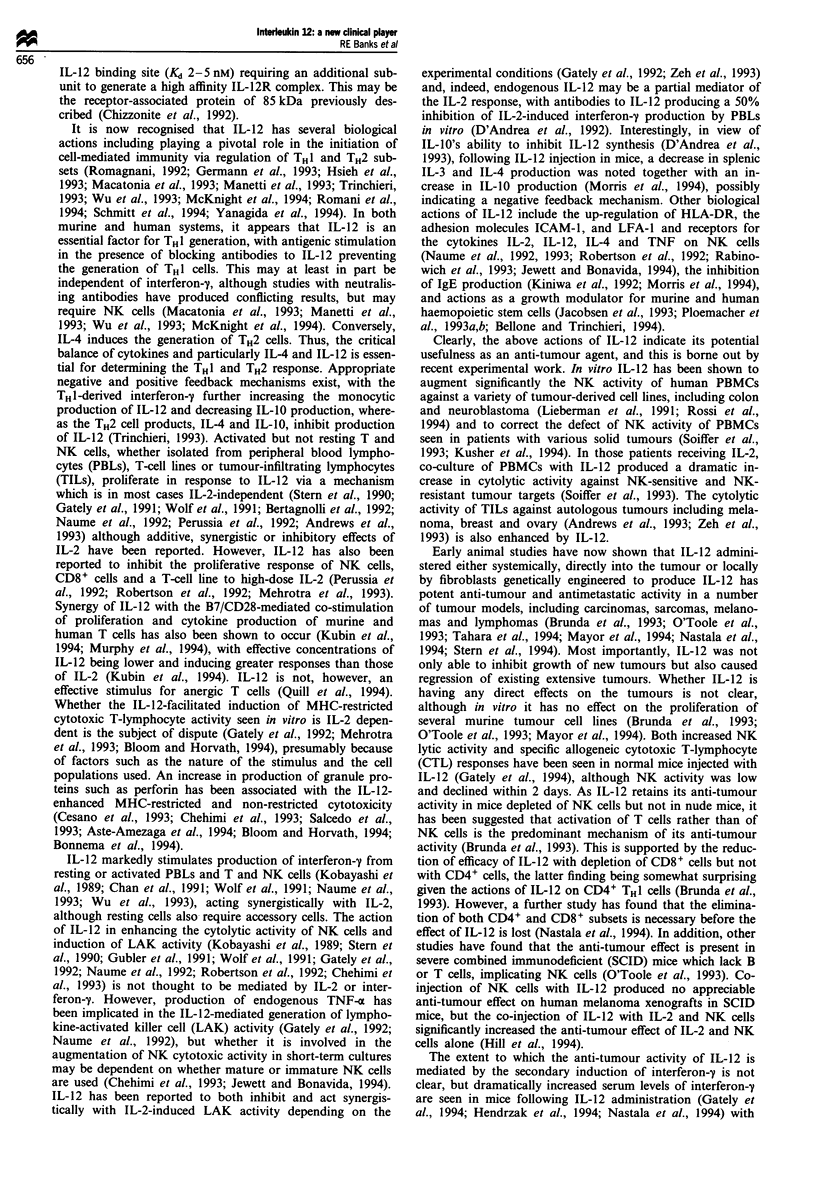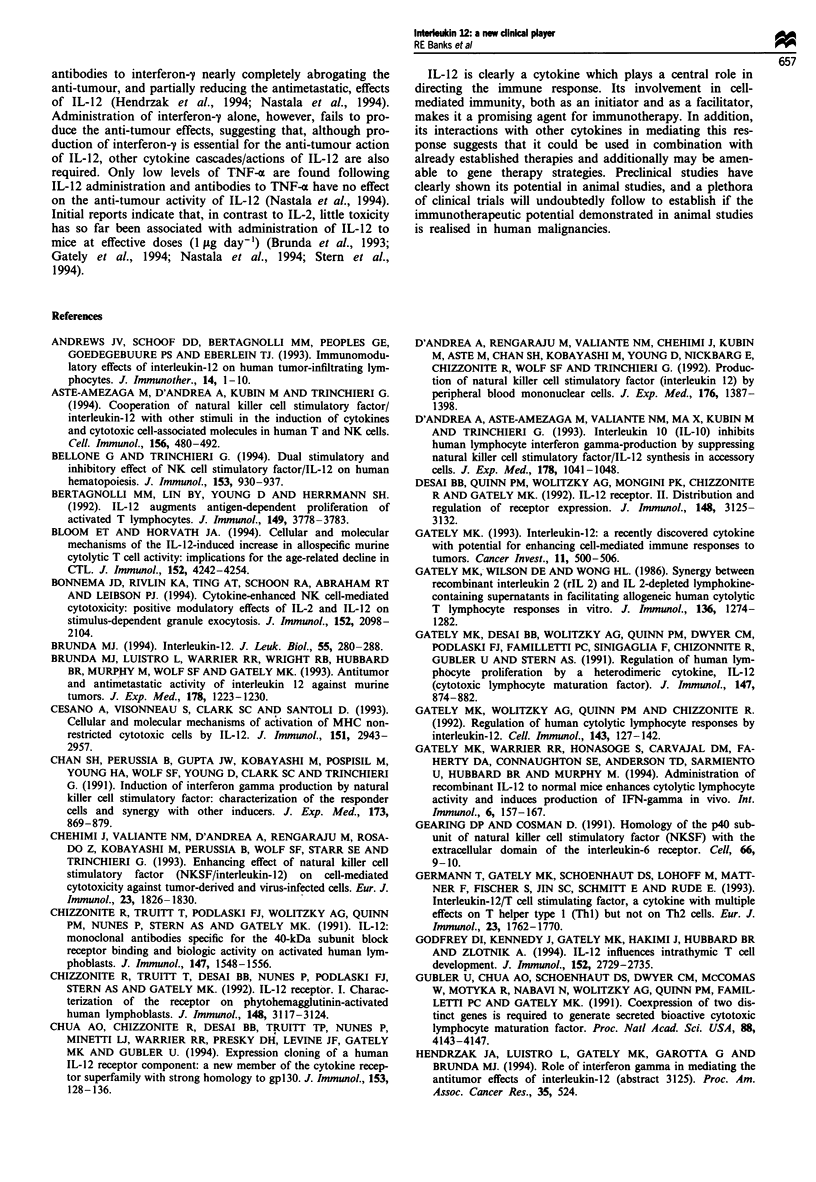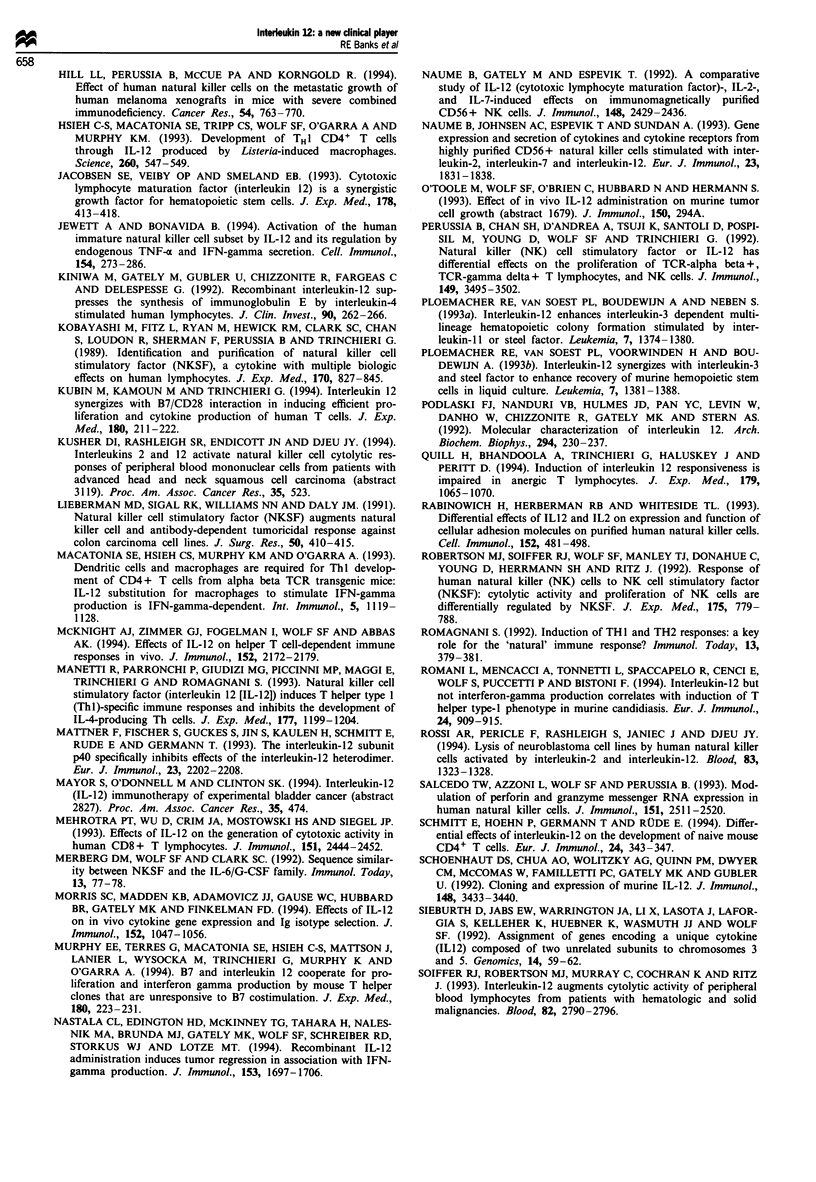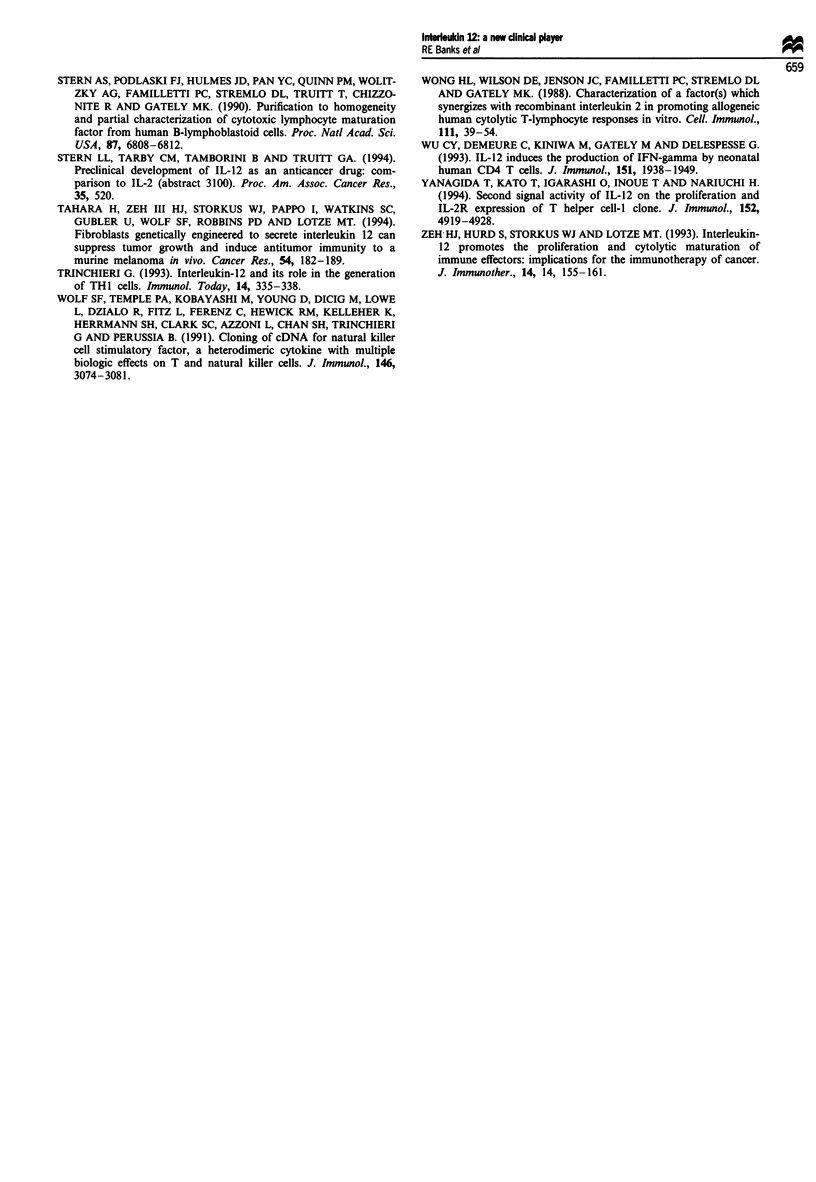# Interleukin 12: a new clinical player in cytokine therapy.

**DOI:** 10.1038/bjc.1995.130

**Published:** 1995-04

**Authors:** R. E. Banks, P. M. Patel, P. J. Selby


British Journal of Cancer (1995) 71, 655–659
© 1995 Stockton Press All rights reserved 0007-0920/95 $12.00

EDITORIAL

### Interleukin 12: a new clinical player in cytokine therapy

MEDICAL LIBRARY
BELLEVUE HOSPITAL

RE Banks, PM Patel and PJ Selby

*ICRF Cancer Medicine Research Unit, St James's University Hospital, Beckett Street, Leeds LS9 7TF, UK.*

**Keywords:** interleukin 12, cytokine, cancer, immunotherapy, review

Interleukin 12 (IL-12) was identified several years ago, but its potential use in cancer therapy has only been recognised relatively recently. Animal studies have now shown that IL-12 has potent anti-tumour and antimetastatic activity, with less toxicity than that encountered with IL-2. In this editorial, we review the literature concerning the biology of IL-12, focusing on the areas of particular relevance to its possible role in cancer therapy. We propose that an important new cytokine has arrived for use in clinical oncology.

IL-12 (reviewed by Gately, 1993; Brunda, 1994) is a 70–75 kDa glycosylated cytokine which was simultaneously identified and purified from the supernatant of human B-lymphoblastoid cell lines by two independent groups and was initially described on the basis of its actions as 'cytotoxic lymphocyte maturation factor (CLMF)' (Gately *et al.*, 1986; Wong *et al.*, 1988; Stern *et al.*, 1990) and 'natural killer cell stimulatory factor (NKSF)' (Kobayashi *et al.*, 1989). It has an atypical structure for a cytokine, being heterodimeric and consisting of a 35 kDa (p35) subunit and a 40 kDa (p40) subunit linked by a disulphide bond. A mixture of soluble recombinant subunits also has biological activity but only at concentrations several orders of magnitude greater than that of the covalently linked heterodimer (Trinchieri, 1993). The genes for both the p35 and p40 subunits have been cloned (Gubler *et al.*, 1991; Wolf *et al.*, 1991) and mapped in humans to chromosomes 3p12–3q13.2 and 5q31–q33 respectively (Sieburth *et al.*, 1992). The predicted amino acid sequences of the human p35 and p40 IL-12 subunits show 60% and 70% identity with the murine sequences and consist of 20% and 10% carbohydrate respectively (Podlaski *et al.*, 1992; Schoenhaut *et al.*, 1992). Bioactivity studies, however, show species specificity, with human IL-12 exhibiting minimal activity in the murine system but with murine IL-12 being active in human systems. The use of interspecies p35/p40 hybrids suggests that this is determined by the p35 subunits (Schoenhaut *et al.*, 1992). However, as antibodies against the p40 subunit can neutralise IL-12 bioactivity, both subunits may be involved in receptor binding (Chizzonite *et al.*, 1991; D'Andrea *et al.*, 1992), either directly or possibly through one subunit influencing the conformational state of the other.

The main cellular sources of IL-12 are monocytes/macrophages, which secrete IL-12 constitutively and particularly following stimulation with bacterially derived products, and B cells and B-lymphoblastoid cell lines (D'Andrea *et al.*, 1992). Incubation of peripheral blood mononuclear cells (PBMCs) with *Staphylococcus aureus* Cowan I strain or lipopolysaccharide (LPS) increases production of IL-12 but a range of cytokines, including IL-1α, IL-1β, IL-2, IL-4, IL-6 and tumour necrosis factor alpha (TNF-α), do not elicit increased production (D'Andrea *et al.*, 1992), and IL-10 has been reported to inhibit synthesis of IL-12 by PBMCs (D'Andrea *et al.*, 1992; Kubin *et al.*, 1994). Discordant production of the subunits has been demonstrated, indicating independent regulation, with excess uncomplexed p40 being produced by human PBMCs and B-lymphoblastoid cell lines, although production of free p35 has not been observed (Stern *et al.*, 1990; Wolf *et al.*, 1991; D'Andrea *et al.*, 1992). A range of solid tumour-derived, T-cell and myeloid leukaemic cell lines have been found not to secrete IL-12, although many of these cell lines contain mRNA transcripts of the p35 gene alone (D'Andrea *et al.*, 1992). Similarly, in the murine system, p40 mRNA has been found to be restricted to lymphoid tissues, whereas p35 mRNA is detected in both lymphoid and non-lymphoid (lung and brain) tissues (Schoenhaut *et al.*, 1992). However, mRNA for both subunits has been found in two murine thymic epithelial lines and a thymic fibroblastoid clone (Godfrey *et al.*, 1994), although it is not known whether these cell types secrete mature IL-12 protein. It is unclear whether the individual IL-12 subunits can exert as yet unidentified biological effects, but the murine p40 subunit is able to inhibit several biological activities of the IL-12 heterodimer (Mattner *et al.*, 1993). This may be analogous to the IL-1 family, in that IL-1α and -β can bind to and elicit responses in target cells, but the third member, the IL-1 receptor antagonist, can bind to the receptor but does not induce a response and can competitively inhibit IL-1α or -β. In effect, the excess of free p40 produced by some cells may serve to regulate the biological activity of IL-12.

A high-affinity 110–135 kDa receptor with a $K_d$ of 100–600 pM has been found on activated human T cells and NK cells but not on B cells, resting PBMCs or several cell lines, including those of T, NK, B, myelomonocytic, epithelial and fibroblast lineage (Chizzonite *et al.*, 1992; Desai *et al.*, 1992). Subsequently, a more detailed analysis has identified three binding sites on phytohaemagglutinin (PHA) activated PBMCs with apparent $K_d$ of 5–20 pM and 2–6 nM (Chua *et al.*, 1994). The extensive homology between the p40 subunit and the extracellular domain of the IL-6 receptor, together with the more distant relationship between the p35 subunit and IL-6 itself (Gearing and Cosman, 1991; Merberg *et al.*, 1992), has provoked speculation that the IL-12 heterodimer may have evolved from a cytokine/soluble receptor complex, such as occurs with IL-6, which then interacts with a cell-bound receptor analogous to gp130 and induces biological activity. This has been given further support by the recent cloning of the cDNA encoding a human IL-12 receptor subunit of approximately 100 kDa which structurally is a member of the haematopoietin receptor family and most closely related to gp130 (Chua *et al.*, 1994). However, unlike the IL-6/soluble IL-6R-induced dimerisation of gp130 and subsequent signal transduction, the oligomerisation of the IL-12R subunit is not dependent on ligand binding and IL-12 only binds dimeric or oligomeric forms of the subunit. Additionally, whereas gp130 expression is widespread, constitutive and converts the IL-6/IL-6R interaction to one of high affinity, the IL-12R subunit is restricted, highly inducible by mitogens or IL-2 and is thought to represent the low-affinity

Correspondence: RE Banks
Received 19 November 1994; revised 23 November 1994; accepted 23 November 1994

IL-12 binding site ($K_d$ 2–5 nM) requiring an additional subunit to generate a high affinity IL-12R complex. This may be the receptor-associated protein of 85 kDa previously described (Chizzonite *et al.*, 1992).

It is now recognised that IL-12 has several biological actions including playing a pivotal role in the initiation of cell-mediated immunity via regulation of $T_H1$ and $T_H2$ subsets (Romagnani, 1992; Germann *et al.*, 1993; Hsieh *et al.*, 1993; Macatonia *et al.*, 1993; Manetti *et al.*, 1993; Trinchieri, 1993; Wu *et al.*, 1993; McKnight *et al.*, 1994; Romani *et al.*, 1994; Schmitt *et al.*, 1994; Yanagida *et al.*, 1994). In both murine and human systems, it appears that IL-12 is an essential factor for $T_H1$ generation, with antigenic stimulation in the presence of blocking antibodies to IL-12 preventing the generation of $T_H1$ cells. This may at least in part be independent of interferon-γ, although studies with neutralising antibodies have produced conflicting results, but may require NK cells (Macatonia *et al.*, 1993; Manetti *et al.*, 1993; Wu *et al.*, 1993; McKnight *et al.*, 1994). Conversely, IL-4 induces the generation of $T_H2$ cells. Thus, the critical balance of cytokines and particularly IL-4 and IL-12 is essential for determining the $T_H1$ and $T_H2$ response. Appropriate negative and positive feedback mechanisms exist, with the $T_H1$-derived interferon-γ further increasing the monocytic production of IL-12 and decreasing IL-10 production, whereas the $T_H2$ cell products, IL-4 and IL-10, inhibit production of IL-12 (Trinchieri, 1993). Activated but not resting T and NK cells, whether isolated from peripheral blood lymphocytes (PBLs), T-cell lines or tumour-infiltrating lymphocytes (TILs), proliferate in response to IL-12 via a mechanism which is in most cases IL-2-independent (Stern *et al.*, 1990; Gately *et al.*, 1991; Wolf *et al.*, 1991; Bertagnolli *et al.*, 1992; Naume *et al.*, 1992; Perussia *et al.*, 1992; Andrews *et al.*, 1993) although additive, synergistic or inhibitory effects of IL-2 have been reported. However, IL-12 has also been reported to inhibit the proliferative response of NK cells, $CD8^+$ cells and a T-cell line to high-dose IL-2 (Perussia *et al.*, 1992; Robertson *et al.*, 1992; Mehrotra *et al.*, 1993). Synergy of IL-12 with the B7/CD28-mediated co-stimulation of proliferation and cytokine production of murine and human T cells has also been shown to occur (Kubin *et al.*, 1994; Murphy *et al.*, 1994), with effective concentrations of IL-12 being lower and inducing greater responses than those of IL-2 (Kubin *et al.*, 1994). IL-12 is not, however, an effective stimulus for anergic T cells (Quill *et al.*, 1994). Whether the IL-12-facilitated induction of MHC-restricted cytotoxic T-lymphocyte activity seen *in vitro* is IL-2 dependent is the subject of dispute (Gately *et al.*, 1992; Mehrotra *et al.*, 1993; Bloom and Horvath, 1994), presumably because of factors such as the nature of the stimulus and the cell populations used. An increase in production of granule proteins such as perforin has been associated with the IL-12-enhanced MHC-restricted and non-restricted cytotoxicity (Cesano *et al.*, 1993; Chehimi *et al.*, 1993; Salcedo *et al.*, 1993; Aste-Amezaga *et al.*, 1994; Bloom and Horvath, 1994; Bonnema *et al.*, 1994).

IL-12 markedly stimulates production of interferon-γ from resting or activated PBLs and T and NK cells (Kobayashi *et al.*, 1989; Chan *et al.*, 1991; Wolf *et al.*, 1991; Naume *et al.*, 1993; Wu *et al.*, 1993), acting synergistically with IL-2, although resting cells also require accessory cells. The action of IL-12 in enhancing the cytolytic activity of NK cells and induction of LAK activity (Kobayashi *et al.*, 1989; Stern *et al.*, 1990; Gubler *et al.*, 1991; Wolf *et al.*, 1991; Gately *et al.*, 1992; Naume *et al.*, 1992; Robertson *et al.*, 1992; Chehimi *et al.*, 1993) is not thought to be mediated by IL-2 or interferon-γ. However, production of endogenous TNF-α has been implicated in the IL-12-mediated generation of lymphokine-activated killer cell (LAK) activity (Gately *et al.*, 1992; Naume *et al.*, 1992), but whether it is involved in the augmentation of NK cytotoxic activity in short-term cultures may be dependent on whether mature or immature NK cells are used (Chehimi *et al.*, 1993; Jewett and Bonavida, 1994). IL-12 has been reported to both inhibit and act synergistically with IL-2-induced LAK activity depending on the experimental conditions (Gately *et al.*, 1992; Zeh *et al.*, 1993) and, indeed, endogenous IL-12 may be a partial mediator of the IL-2 response, with antibodies to IL-12 producing a 50% inhibition of IL-2-induced interferon-γ production by PBLs *in vitro* (D'Andrea *et al.*, 1992). Interestingly, in view of IL-10's ability to inhibit IL-12 synthesis (D'Andrea *et al.*, 1993), following IL-12 injection in mice, a decrease in splenic IL-3 and IL-4 production was noted together with an increase in IL-10 production (Morris *et al.*, 1994), possibly indicating a negative feedback mechanism. Other biological actions of IL-12 include the up-regulation of HLA-DR, the adhesion molecules ICAM-1, and LFA-1 and receptors for the cytokines IL-2, IL-12, IL-4 and TNF on NK cells (Naume *et al.*, 1992, 1993; Robertson *et al.*, 1992; Rabinowich *et al.*, 1993; Jewett and Bonavida, 1994), the inhibition of IgE production (Kiniwa *et al.*, 1992; Morris *et al.*, 1994), and actions as a growth modulator for murine and human haemopoietic stem cells (Jacobsen *et al.*, 1993; Ploemacher *et al.*, 1993*a*,*b*; Bellone and Trinchieri, 1994).

Clearly, the above actions of IL-12 indicate its potential usefulness as an anti-tumour agent, and this is borne out by recent experimental work. *In vitro* IL-12 has been shown to augment significantly the NK activity of human PBMCs against a variety of tumour-derived cell lines, including colon and neuroblastoma (Lieberman *et al.*, 1991; Rossi *et al.*, 1994) and to correct the defect of NK activity of PBMCs seen in patients with various solid tumours (Soiffer *et al.*, 1993; Kusher *et al.*, 1994). In those patients receiving IL-2, co-culture of PBMCs with IL-12 produced a dramatic increase in cytolytic activity against NK-sensitive and NK-resistant tumour targets (Soiffer *et al.*, 1993). The cytolytic activity of TILs against autologous tumours including melanoma, breast and ovary (Andrews *et al.*, 1993; Zeh *et al.*, 1993) is also enhanced by IL-12.

Early animal studies have now shown that IL-12 administered either systemically, directly into the tumour or locally by fibroblasts genetically engineered to produce IL-12 has potent anti-tumour and antimetastatic activity in a number of tumour models, including carcinomas, sarcomas, melanomas and lymphomas (Brunda *et al.*, 1993; O'Toole *et al.*, 1993; Tahara *et al.*, 1994; Mayor *et al.*, 1994; Nastala *et al.*, 1994; Stern *et al.*, 1994). Most importantly, IL-12 was not only able to inhibit growth of new tumours but also caused regression of existing extensive tumours. Whether IL-12 is having any direct effects on the tumours is not clear, although *in vitro* it has no effect on the proliferation of several murine tumour cell lines (Brunda *et al.*, 1993; O'Toole *et al.*, 1993; Mayor *et al.*, 1994). Both increased NK lytic activity and specific allogeneic cytotoxic T-lymphocyte (CTL) responses have been seen in normal mice injected with IL-12 (Gately *et al.*, 1994), although NK activity was low and declined within 2 days. As IL-12 retains its anti-tumour activity in mice depleted of NK cells but not in nude mice, it has been suggested that activation of T cells rather than of NK cells is the predominant mechanism of its anti-tumour activity (Brunda *et al.*, 1993). This is supported by the reduction of efficacy of IL-12 with depletion of $CD8^+$ cells but not with $CD4^+$ cells, the latter finding being somewhat surprising given the actions of IL-12 on $CD4^+$ $T_H1$ cells (Brunda *et al.*, 1993). However, a further study has found that the elimination of both $CD4^+$ and $CD8^+$ subsets is necessary before the effect of IL-12 is lost (Nastala *et al.*, 1994). In addition, other studies have found that the anti-tumour effect is present in severe combined immunodeficient (SCID) mice which lack B or T cells, implicating NK cells (O'Toole *et al.*, 1993). Co-injection of NK cells with IL-12 produced no appreciable anti-tumour effect on human melanoma xenografts in SCID mice, but the co-injection of IL-12 with IL-2 and NK cells significantly increased the anti-tumour effect of IL-2 and NK cells alone (Hill *et al.*, 1994).

The extent to which the anti-tumour activity of IL-12 is mediated by the secondary induction of interferon-γ is not clear, but dramatically increased serum levels of interferon-γ are seen in mice following IL-12 administration (Gately *et al.*, 1994; Hendrzak *et al.*, 1994; Nastala *et al.*, 1994) with antibodies to interferon-γ nearly completely abrogating the anti-tumour, and partially reducing the antimetastatic, effects of IL-12 (Hendrzak *et al.*, 1994; Nastala *et al.*, 1994). Administration of interferon-γ alone, however, fails to produce the anti-tumour effects, suggesting that, although production of interferon-γ is essential for the anti-tumour action of IL-12, other cytokine cascades/actions of IL-12 are also required. Only low levels of TNF-α are found following IL-12 administration and antibodies to TNF-α have no effect on the anti-tumour activity of IL-12 (Nastala *et al.*, 1994). Initial reports indicate that, in contrast to IL-2, little toxicity has so far been associated with administration of IL-12 to mice at effective doses (1 μg $day^{-1}$) (Brunda *et al.*, 1993; Gately *et al.*, 1994; Nastala *et al.*, 1994; Stern *et al.*, 1994).

IL-12 is clearly a cytokine which plays a central role in directing the immune response. Its involvement in cell-mediated immunity, both as an initiator and as a facilitator, makes it a promising agent for immunotherapy. In addition, its interactions with other cytokines in mediating this response suggests that it could be used in combination with already established therapies and additionally may be amenable to gene therapy strategies. Preclinical studies have clearly shown its potential in animal studies, and a plethora of clinical trials will undoubtedly follow to establish if the immunotherapeutic potential demonstrated in animal studies is realised in human malignancies.

### References

ANDREWS JV, SCHOOF DD, BERTAGNOLLI MM, PEOPLES GE, GOEDEGEBUURE PS AND EBERLEIN TJ. (1993). Immunomodulatory effects of interleukin-12 on human tumor-infiltrating lymphocytes. *J. Immunother.*, **14**, 1–10.

ASTE-AMEZAGA M, D'ANDREA A, KUBIN M AND TRINCHIERI G. (1994). Cooperation of natural killer cell stimulatory factor/interleukin-12 with other stimuli in the induction of cytokines and cytotoxic cell-associated molecules in human T and NK cells. *Cell. Immunol.*, **156**, 480–492.

BELLONE G AND TRINCHIERI G. (1994). Dual stimulatory and inhibitory effect of NK cell stimulatory factor/IL-12 on human hematopoiesis. *J. Immunol.*, **153**, 930–937.

BERTAGNOLLI MM, LIN BY, YOUNG D AND HERRMANN SH. (1992). IL-12 augments antigen-dependent proliferation of activated T lymphocytes. *J. Immunol.*, **149**, 3778–3783.

BLOOM ET AND HORVATH JA. (1994). Cellular and molecular mechanisms of the IL-12-induced increase in allospecific murine cytolytic T cell activity: implications for the age-related decline in CTL. *J. Immunol.*, **152**, 4242–4254.

BONNEMA JD, RIVLIN KA, TING AT, SCHOON RA, ABRAHAM RT AND LEIBSON PJ. (1994). Cytokine-enhanced NK cell-mediated cytotoxicity: positive modulatory effects of IL-2 and IL-12 on stimulus-dependent granule exocytosis. *J. Immunol.*, **152**, 2098–2104.

BRUNDA MJ. (1994). Interleukin-12. *J. Leuk. Biol.*, **55**, 280–288.

BRUNDA MJ, LUISTRO L, WARRIER RR, WRIGHT RB, HUBBARD BR, MURPHY M, WOLF SF AND GATELY MK. (1993). Antitumor and antimetastatic activity of interleukin 12 against murine tumors. *J. Exp. Med.*, **178**, 1223–1230.

CESANO A, VISONNEAU S, CLARK SC AND SANTOLI D. (1993). Cellular and molecular mechanisms of activation of MHC non-restricted cytotoxic cells by IL-12. *J. Immunol.*, **151**, 2943–2957.

CHAN SH, PERUSSIA B, GUPTA JW, KOBAYASHI M, POSPISIL M, YOUNG HA, WOLF SF, YOUNG D, CLARK SC AND TRINCHIERI G. (1991). Induction of interferon gamma production by natural killer cell stimulatory factor: characterization of the responder cells and synergy with other inducers. *J. Exp. Med.*, **173**, 869–879.

CHEHIMI J, VALIANTE NM, D'ANDREA A, RENGARAJU M, ROSADO Z, KOBAYASHI M, PERUSSIA B, WOLF SF, STARR SE AND TRINCHIERI G. (1993). Enhancing effect of natural killer cell stimulatory factor (NKSF/interleukin-12) on cell-mediated cytotoxicity against tumor-derived and virus-infected cells. *Eur. J. Immunol.*, **23**, 1826–1830.

CHIZZONITE R, TRUITT T, PODLASKI FJ, WOLITZKY AG, QUINN PM, NUNES P, STERN AS AND GATELY MK. (1991). IL-12: monoclonal antibodies specific for the 40-kDa subunit block receptor binding and biologic activity on activated human lymphoblasts. *J. Immunol.*, **147**, 1548–1556.

CHIZZONITE R, TRUITT T, DESAI BB, NUNES P, PODLASKI FJ, STERN AS AND GATELY MK. (1992). IL-12 receptor. I. Characterization of the receptor on phytohemagglutinin-activated human lymphoblasts. *J. Immunol.*, **148**, 3117–3124.

CHUA AO, CHIZZONITE R, DESAI BB, TRUITT TP, NUNES P, MINETTI LJ, WARRIER RR, PRESKY DH, LEVINE JF, GATELY MK AND GUBLER U. (1994). Expression cloning of a human IL-12 receptor component: a new member of the cytokine receptor superfamily with strong homology to gp130. *J. Immunol.*, **153**, 128–136.

D'ANDREA A, RENGARAJU M, VALIANTE NM, CHEHIMI J, KUBIN M, ASTE M, CHAN SH, KOBAYASHI M, YOUNG D, NICKBARG E, CHIZZONITE R, WOLF SF AND TRINCHIERI G. (1992). Production of natural killer cell stimulatory factor (interleukin 12) by peripheral blood mononuclear cells. *J. Exp. Med.*, **176**, 1387–1398.

D'ANDREA A, ASTE-AMEZAGA M, VALIANTE NM, MA X, KUBIN M AND TRINCHIERI G. (1993). Interleukin 10 (IL-10) inhibits human lymphocyte interferon gamma-production by suppressing natural killer cell stimulatory factor/IL-12 synthesis in accessory cells. *J. Exp. Med.*, **178**, 1041–1048.

DESAI BB, QUINN PM, WOLITZKY AG, MONGINI PK, CHIZZONITE R AND GATELY MK. (1992). IL-12 receptor. II. Distribution and regulation of receptor expression. *J. Immunol.*, **148**, 3125–3132.

GATELY MK. (1993). Interleukin-12: a recently discovered cytokine with potential for enhancing cell-mediated immune responses to tumors. *Cancer Invest.*, **11**, 500–506.

GATELY MK, WILSON DE AND WONG HL. (1986). Synergy between recombinant interleukin 2 (rIL 2) and IL 2-depleted lymphokine-containing supernatants in facilitating allogeneic human cytolytic T lymphocyte responses in vitro. *J. Immunol.*, **136**, 1274–1282.

GATELY MK, DESAI BB, WOLITZKY AG, QUINN PM, DWYER CM, PODLASKI FJ, FAMILLETTI PC, SINIGAGLIA F, CHIZONNITE R, GUBLER U AND STERN AS. (1991). Regulation of human lymphocyte proliferation by a heterodimeric cytokine, IL-12 (cytotoxic lymphocyte maturation factor). *J. Immunol.*, **147**, 874–882.

GATELY MK, WOLITZKY AG, QUINN PM AND CHIZZONITE R. (1992). Regulation of human cytolytic lymphocyte responses by interleukin-12. *Cell. Immunol.*, **143**, 127–142.

GATELY MK, WARRIER RR, HONASOGE S, CARVAJAL DM, FAHERTY DA, CONNAUGHTON SE, ANDERSON TD, SARMIENTO U, HUBBARD BR AND MURPHY M. (1994). Administration of recombinant IL-12 to normal mice enhances cytolytic lymphocyte activity and induces production of IFN-gamma in vivo. *Int. Immunol.*, **6**, 157–167.

GEARING DP AND COSMAN D. (1991). Homology of the p40 subunit of natural killer cell stimulatory factor (NKSF) with the extracellular domain of the interleukin-6 receptor. *Cell*, **66**, 9–10.

GERMANN T, GATELY MK, SCHOENHAUT DS, LOHOFF M, MATTNER F, FISCHER S, JIN SC, SCHMITT E AND RUDE E. (1993). Interleukin-12/T cell stimulating factor, a cytokine with multiple effects on T helper type 1 (Th1) but not on Th2 cells. *Eur. J. Immunol.*, **23**, 1762–1770.

GODFREY DI, KENNEDY J, GATELY MK, HAKIMI J, HUBBARD BR AND ZLOTNIK A. (1994). IL-12 influences intrathymic T cell development. *J. Immunol.*, **152**, 2729–2735.

GUBLER U, CHUA AO, SCHOENHAUT DS, DWYER CM, MCCOMAS W, MOTYKA R, NABAVI N, WOLITZKY AG, QUINN PM, FAMILLETTI PC AND GATELY MK. (1991). Coexpression of two distinct genes is required to generate secreted bioactive cytotoxic lymphocyte maturation factor. *Proc. Natl Acad. Sci. USA*, **88**, 4143–4147.

HENDRZAK JA, LUISTRO L, GATELY MK, GAROTTA G AND BRUNDA MJ. (1994). Role of interferon gamma in mediating the antitumor effects of interleukin-12 (abstract 3125). *Proc. Am. Assoc. Cancer Res.*, **35**, 524.

HILL LL, PERUSSIA B, MCCUE PA AND KORNGOLD R. (1994). Effect of human natural killer cells on the metastatic growth of human melanoma xenografts in mice with severe combined immunodeficiency. *Cancer Res.*, **54**, 763–770.

HSIEH C-S, MACATONIA SE, TRIPP CS, WOLF SF, O'GARRA A AND MURPHY KM. (1993). Development of $T_H1$ $CD4^+$ T cells through IL-12 produced by *Listeria*-induced macrophages. *Science*, **260**, 547–549.

JACOBSEN SE, VEIBY OP AND SMELAND EB. (1993). Cytotoxic lymphocyte maturation factor (interleukin 12) is a synergistic growth factor for hematopoietic stem cells. *J. Exp. Med.*, **178**, 413–418.

JEWETT A AND BONAVIDA B. (1994). Activation of the human immature natural killer cell subset by IL-12 and its regulation by endogenous TNF-α and IFN-gamma secretion. *Cell. Immunol.*, **154**, 273–286.

KINIWA M, GATELY M, GUBLER U, CHIZZONITE R, FARGEAS C AND DELESPESSE G. (1992). Recombinant interleukin-12 suppresses the synthesis of immunoglobulin E by interleukin-4 stimulated human lymphocytes. *J. Clin. Invest.*, **90**, 262–266.

KOBAYASHI M, FITZ L, RYAN M, HEWICK RM, CLARK SC, CHAN S, LOUDON R, SHERMAN F, PERUSSIA B AND TRINCHIERI G. (1989). Identification and purification of natural killer cell stimulatory factor (NKSF), a cytokine with multiple biologic effects on human lymphocytes. *J. Exp. Med.*, **170**, 827–845.

KUBIN M, KAMOUN M AND TRINCHIERI G. (1994). Interleukin 12 synergizes with B7/CD28 interaction in inducing efficient proliferation and cytokine production of human T cells. *J. Exp. Med.*, **180**, 211–222.

KUSHER DI, RASHLEIGH SR, ENDICOTT JN AND DJEU JY. (1994). Interleukins 2 and 12 activate natural killer cell cytolytic responses of peripheral blood mononuclear cells from patients with advanced head and neck squamous cell carcinoma (abstract 3119). *Proc. Am. Assoc. Cancer Res.*, **35**, 523.

LIEBERMAN MD, SIGAL RK, WILLIAMS NN AND DALY JM. (1991). Natural killer cell stimulatory factor (NKSF) augments natural killer cell and antibody-dependent tumoricidal response against colon carcinoma cell lines. *J. Surg. Res.*, **50**, 410–415.

MACATONIA SE, HSIEH CS, MURPHY KM AND O'GARRA A. (1993). Dendritic cells and macrophages are required for Th1 development of CD4+ T cells from alpha beta TCR transgenic mice: IL-12 substitution for macrophages to stimulate IFN-gamma production is IFN-gamma-dependent. *Int. Immunol.*, **5**, 1119–1128.

MCKNIGHT AJ, ZIMMER GJ, FOGELMAN I, WOLF SF AND ABBAS AK. (1994). Effects of IL-12 on helper T cell-dependent immune responses in vivo. *J. Immunol.*, **152**, 2172–2179.

MANETTI R, PARRONCHI P, GIUDIZI MG, PICCINNI MP, MAGGI E, TRINCHIERI G AND ROMAGNANI S. (1993). Natural killer cell stimulatory factor (interleukin 12 [IL-12]) induces T helper type 1 (Th1)-specific immune responses and inhibits the development of IL-4-producing Th cells. *J. Exp. Med.*, **177**, 1199–1204.

MATTNER F, FISCHER S, GUCKES S, JIN S, KAULEN H, SCHMITT E, RUDE E AND GERMANN T. (1993). The interleukin-12 subunit p40 specifically inhibits effects of the interleukin-12 heterodimer. *Eur. J. Immunol.*, **23**, 2202–2208.

MAYOR S, O'DONNELL M AND CLINTON SK. (1994). Interleukin-12 (IL-12) immunotherapy of experimental bladder cancer (abstract 2827). *Proc. Am. Assoc. Cancer Res.*, **35**, 474.

MEHROTRA PT, WU D, CRIM JA, MOSTOWSKI HS AND SIEGEL JP. (1993). Effects of IL-12 on the generation of cytotoxic activity in human CD8+ T lymphocytes. *J. Immunol.*, **151**, 2444–2452.

MERBERG DM, WOLF SF AND CLARK SC. (1992). Sequence similarity between NKSF and the IL-6/G-CSF family. *Immunol. Today*, **13**, 77–78.

MORRIS SC, MADDEN KB, ADAMOVICZ JJ, GAUSE WC, HUBBARD BR, GATELY MK AND FINKELMAN FD. (1994). Effects of IL-12 on in vivo cytokine gene expression and Ig isotype selection. *J. Immunol.*, **152**, 1047–1056.

MURPHY EE, TERRES G, MACATONIA SE, HSIEH C-S, MATTSON J, LANIER L, WYSOCKA M, TRINCHIERI G, MURPHY K AND O'GARRA A. (1994). B7 and interleukin 12 cooperate for proliferation and interferon gamma production by mouse T helper clones that are unresponsive to B7 costimulation. *J. Exp. Med.*, **180**, 223–231.

NASTALA CL, EDINGTON HD, MCKINNEY TG, TAHARA H, NALESNIK MA, BRUNDA MJ, GATELY MK, WOLF SF, SCHREIBER RD, STORKUS WJ AND LOTZE MT. (1994). Recombinant IL-12 administration induces tumor regression in association with IFN-gamma production. *J. Immunol.*, **153**, 1697–1706.

NAUME B, GATELY M AND ESPEVIK T. (1992). A comparative study of IL-12 (cytotoxic lymphocyte maturation factor)-, IL-2-, and IL-7-induced effects on immunomagnetically purified CD56+ NK cells. *J. Immunol.*, **148**, 2429–2436.

NAUME B, JOHNSEN AC, ESPEVIK T AND SUNDAN A. (1993). Gene expression and secretion of cytokines and cytokine receptors from highly purified CD56+ natural killer cells stimulated with interleukin-2, interleukin-7 and interleukin-12. *Eur. J. Immunol.*, **23**, 1831–1838.

O'TOOLE M, WOLF SF, O'BRIEN C, HUBBARD N AND HERMANN S. (1993). Effect of in vivo IL-12 administration on murine tumor cell growth (abstract 1679). *J. Immunol.*, **150**, 294A.

PERUSSIA B, CHAN SH, D'ANDREA A, TSUJI K, SANTOLI D, POSPISIL M, YOUNG D, WOLF SF AND TRINCHIERI G. (1992). Natural killer (NK) cell stimulatory factor or IL-12 has differential effects on the proliferation of TCR-alpha beta+, TCR-gamma delta+ T lymphocytes, and NK cells. *J. Immunol.*, **149**, 3495–3502.

PLOEMACHER RE, VAN SOEST PL, BOUDEWIJN A AND NEBEN S. (1993*a*). Interleukin-12 enhances interleukin-3 dependent multilineage hematopoietic colony formation stimulated by interleukin-11 or steel factor. *Leukemia*, **7**, 1374–1380.

PLOEMACHER RE, VAN SOEST PL, VOORWINDEN H AND BOUDEWIJN A. (1993*b*). Interleukin-12 synergizes with interleukin-3 and steel factor to enhance recovery of murine hemopoietic stem cells in liquid culture. *Leukemia*, **7**, 1381–1388.

PODLASKI FJ, NANDURI VB, HULMES JD, PAN YC, LEVIN W, DANHO W, CHIZZONITE R, GATELY MK AND STERN AS. (1992). Molecular characterization of interleukin 12. *Arch. Biochem. Biophys.*, **294**, 230–237.

QUILL H, BHANDOOLA A, TRINCHIERI G, HALUSKEY J AND PERITT D. (1994). Induction of interleukin 12 responsiveness is impaired in anergic T lymphocytes. *J. Exp. Med.*, **179**, 1065–1070.

RABINOWICH H, HERBERMAN RB AND WHITESIDE TL. (1993). Differential effects of IL12 and IL2 on expression and function of cellular adhesion molecules on purified human natural killer cells. *Cell. Immunol.*, **152**, 481–498.

ROBERTSON MJ, SOIFFER RJ, WOLF SF, MANLEY TJ, DONAHUE C, YOUNG D, HERRMANN SH AND RITZ J. (1992). Response of human natural killer (NK) cells to NK cell stimulatory factor (NKSF): cytolytic activity and proliferation of NK cells are differentially regulated by NKSF. *J. Exp. Med.*, **175**, 779–788.

ROMAGNANI S. (1992). Induction of TH1 and TH2 responses: a key role for the 'natural' immune response? *Immunol. Today*, **13**, 379–381.

ROMANI L, MENCACCI A, TONNETTI L, SPACCAPELO R, CENCI E, WOLF S, PUCCETTI P AND BISTONI F. (1994). Interleukin-12 but not interferon-gamma production correlates with induction of T helper type-1 phenotype in murine candidiasis. *Eur. J. Immunol.*, **24**, 909–915.

ROSSI AR, PERICLE F, RASHLEIGH S, JANIEC J AND DJEU JY. (1994). Lysis of neuroblastoma cell lines by human natural killer cells activated by interleukin-2 and interleukin-12. *Blood*, **83**, 1323–1328.

SALCEDO TW, AZZONI L, WOLF SF AND PERUSSIA B. (1993). Modulation of perforin and granzyme messenger RNA expression in human natural killer cells. *J. Immunol.*, **151**, 2511–2520.

SCHMITT E, HOEHN P, GERMANN T AND RÜDE E. (1994). Differential effects of interleukin-12 on the development of naive mouse $CD4^+$ T cells. *Eur. J. Immunol.*, **24**, 343–347.

SCHOENHAUT DS, CHUA AO, WOLITZKY AG, QUINN PM, DWYER CM, MCCOMAS W, FAMILLETTI PC, GATELY MK AND GUBLER U. (1992). Cloning and expression of murine IL-12. *J. Immunol.*, **148**, 3433–3440.

SIEBURTH D, JABS EW, WARRINGTON JA, LI X, LASOTA J, LAFORGIA S, KELLEHER K, HUEBNER K, WASMUTH JJ AND WOLF SF. (1992). Assignment of genes encoding a unique cytokine (IL12) composed of two unrelated subunits to chromosomes 3 and 5. *Genomics*, **14**, 59–62.

SOIFFER RJ, ROBERTSON MJ, MURRAY C, COCHRAN K AND RITZ J. (1993). Interleukin-12 augments cytolytic activity of peripheral blood lymphocytes from patients with hematologic and solid malignancies. *Blood*, **82**, 2790–2796.

STERN AS, PODLASKI FJ, HULMES JD, PAN YC, QUINN PM, WOLITZKY AG, FAMILLETTI PC, STREMLO DL, TRUITT T, CHIZZONITE R AND GATELY MK. (1990). Purification to homogeneity and partial characterization of cytotoxic lymphocyte maturation factor from human B-lymphoblastoid cells. *Proc. Natl Acad. Sci. USA*, **87**, 6808–6812.

STERN LL, TARBY CM, TAMBORINI B AND TRUITT GA. (1994). Preclinical development of IL-12 as an anticancer drug: comparison to IL-2 (abstract 3100). *Proc. Am. Assoc. Cancer Res.*, **35**, 520.

TAHARA H, ZEH III HJ, STORKUS WJ, PAPPO I, WATKINS SC, GUBLER U, WOLF SF, ROBBINS PD AND LOTZE MT. (1994). Fibroblasts genetically engineered to secrete interleukin 12 can suppress tumor growth and induce antitumor immunity to a murine melanoma *in vivo*. *Cancer Res.*, **54**, 182–189.

TRINCHIERI G. (1993). Interleukin-12 and its role in the generation of TH1 cells. *Immunol. Today*, **14**, 335–338.

WOLF SF, TEMPLE PA, KOBAYASHI M, YOUNG D, DICIG M, LOWE L, DZIALO R, FITZ L, FERENZ C, HEWICK RM, KELLEHER K, HERRMANN SH, CLARK SC, AZZONI L, CHAN SH, TRINCHIERI G AND PERUSSIA B. (1991). Cloning of cDNA for natural killer cell stimulatory factor, a heterodimeric cytokine with multiple biologic effects on T and natural killer cells. *J. Immunol.*, **146**, 3074–3081.

WONG HL, WILSON DE, JENSON JC, FAMILLETTI PC, STREMLO DL AND GATELY MK. (1988). Characterization of a factor(s) which synergizes with recombinant interleukin 2 in promoting allogeneic human cytolytic T-lymphocyte responses in vitro. *Cell. Immunol.*, **111**, 39–54.

WU CY, DEMEURE C, KINIWA M, GATELY M AND DELESPESSE G. (1993). IL-12 induces the production of IFN-gamma by neonatal human CD4 T cells. *J. Immunol.*, **151**, 1938–1949.

YANAGIDA T, KATO T, IGARASHI O, INOUE T AND NARIUCHI H. (1994). Second signal activity of IL-12 on the proliferation and IL-2R expression of T helper cell-1 clone. *J. Immunol.*, **152**, 4919–4928.

ZEH HJ, HURD S, STORKUS WJ AND LOTZE MT. (1993). Interleukin-12 promotes the proliferation and cytolytic maturation of immune effectors: implications for the immunotherapy of cancer. *J. Immunother.*, **14**, 14, 155–161.